# High incidence of acute kidney injury among patients with major trauma at Mulago National Referral Hospital, Uganda: risk factors and overall survival

**DOI:** 10.4314/ahs.v22i4.23

**Published:** 2022-12

**Authors:** Badru Ssekitooleko, Bashir Ssuna, Stella Alice Nimanya, Ronald Kiwewa, Yasin Ssewanyana, Emmanuel Nkonge, Emmanuel Bua, Joel Wandabwa, William Ocen, Rosemary Nassanga, Frank Asiimwe, Robert Kalyesubula

**Affiliations:** 1 Makerere University College of Health Sciences, School of Medicine; 2 Department of Surgery, Mulago National Referral Hospital; 3 Department of Epidemiology and Biostatistics, College of Health Sciences, Makerere University; 4 Uganda TB Implementation Research Consortium; 5 Islamic University in Uganda

**Keywords:** AKI=Acute kidney injury, major trauma, ISS = injury severity score

## Abstract

**Introduction:**

Acute kidney injury (AKI) is a common and life-threatening complication of major trauma. Recognition is often delayed and management is frequently sub-optimal. We determined the incidence, risk factors and immediate outcomes of AKI in patients with major trauma at Mulago National Referral Hospital.

**Methods:**

This was a prospective study. We recruited adult patients with ISS of > 16. The KDIGO criteria was used to stage AKI. Serum creatinine was measured at baseline, 24, 48, 72 hours and on discharge from the study. Participants were followed up for seven days if not yet discharged. Bivariate and multivariate analysis was done using modified Poisson regression with robust standard errors.

**Results:**

224 patients were recruited. The incidence was 67/1000 persons per day. The risk factors were male sex, delayed presentation, hypoglycemia at admission, RR=1.62 (95%CI 1.24–2.12) and non-operative management RR=1.39 (95%CI 1.02–1.89). Out of the 62 patients that died, 34 (54.8%) had AKI. The overall mortality rate was 39.5 patients per thousand per day.

**Conclusion:**

There was a high incidence of AKI among patients with major trauma. Efforts to reduce morbidity and mortality should be prioritized.

## Introduction

Acute kidney injury (AKI) refers to an abrupt deceleration in kidney function resulting in the retention of urea and other nitrogenous waste products and dysregulation of extracellular volume and electrolytes^5^. The Kidney Disease: Improving Global Outcomes (KDIGO), the most recent and preferred staging guidelines define AKI as increase in serum creatinine by ≥0.3 mg/dL (≥26.5 micromol/L) within 48 hours, or increase in serum creatinine to ≥1.5 times baseline, which is known or presumed to have occurred within the prior seven days, or urine volume <0.5 mL/kg/hour for six hours^5^,^6^. Worldwide, about 16,000 people die every day as a result of major trauma^1^. The latter is defined by the injury severity criteria as a score greater than fifteen^2^. The injury severity score (ISS) is defined as the sum of squares of the highest abbreviated injury score (AIS) grade in the 3 most severely injured body regions that is to say; the chest, abdomen, head and neck, face, bony pelvis and extremities, and external structures^3^,^4^.

The prevalence of AKI in major trauma is appreciably high standing 17.3% to 40.3%, with the commonest risk factors noted being traumatic brain injury, Glasgow Coma scale < 10, ISS > 16^2^,^7^. However, a higher prevalence of 50% was reported in 436 hospitalized trauma patients^8^. Other notable risk factors of AKI post trauma include, use of drugs like furosemide, elevated intra-abdominal pressure, old age, female gender, hypotension, rhabdomyolysis, and the presence of co-morbidities^7^,^9^,^10^. In Mulago Hospital, 6000 injured patients are managed every year, making trauma the single most common reason for admission to the surgical wards with an estimated mortality of 29%^11^,^12^. Acute kidney injury (AKI) has been reported to contribute a mortality rate as high as 30% in patients with major trauma^8^ and yet its incidence was yet to be determined in our setting. This study therefore aimed to determine the incidence, risk factors and immediate outcomes of acute kidney injury among patients with major trauma at Mulago Hospital.

Understanding AKI among severely injured patients will highlight the burden of this problem such that policy guidelines on prevention and management of the severely injured patients prone to AKI can be drawn. Dialysis is highly inaccessible in Mulago National referral Hospital for most of the patients with chronic kidney disease further augmenting the need for prevention of AKI.

## Methods

### Study design

This was a prospective cohort study.

### Study setting

The study was conducted in Mulago National Referral Hospital which is located in the northern part of Kampala capital city, immediately west of the Makerere University College of Health Sciences. Patients were recruited from the Accident and Emergency (A&E) unit and there followed up from the respective inpatient wards.

### Study population

We recruited all patients above 17 years of age with major trauma who presented to the A and E department from January 2018 to May 2018 who met the eligibility criteria.

### Eligibility criteria

#### Inclusion criteria

- Must have given an informed consent.
- Trauma patients with an ISS Score of >16

#### Exclusion criteria

- Referred trauma patients after receiving any form of treatment.
- Failure to get consent from the patient or treatment supporter.

### Sample size

We used Kish Leslie formula (1965) for a single proportion using a study by Bagshawel et al, 2008 (P=18.1%)^9^ which gave 224 participants and a formula of two proportions for the risk factors using results reported by de Abreu KLS et al, 20107and we got a sample size of 197.

### Sampling procedure

Consecutive sampling was used throughout the study as participants presented to A&E unit to include only those meeting our eligibility criteria.

### Study variables

#### Independent variables

**These included:** Age, Sex, Mechanism of injury, Body region injured, Duration of injury, Co-morbidities like DM, HT, and HIV, ISS score and drugs used.

#### Dependent variable

Acute kidney injury in major trauma.

### Study procedure

Patients were initially scored using the Abbreviated Injury score (AIS). The Injury Severity Score (ISS) was then calculated as the sum of squares of the highest AIS grade in the 3 most severely injured body regions. On a given day of data collection, all Patients admitted at Accident and Emergency unit were screened. Patients and their care takers were asked if any treatment was received from another facility before coming to MNRH, those that answered in affirmative were excluded. Those with an ISS > 16 and fitting into the inclusion criteria were then recruited randomly.

Serum creatinine was measured on admission and then repeated at 24, 48, 72 hours and upon discharge from the study.

### Specimen collection, storage, and handling procedures

About 1–2 ml of venous blood was drawn from the patient, and immediately transported to the A and E chemistry laboratory and analysis for creatinine was done. Specimens that were not analysed immediately were refrigerated at < –20°C.

### Data collection tool

An interviewer administered questionnaire translated in the local language (Luganda) was used to collect data. KDIGO criteria was used to classify AKI.

### Data management and analysis

All data captured was entered into an electronic Epidata version 3.1. The database was reviewed for consistency and completeness. After cleaning, the dataset was exported to Stata Version 15.0/MP for analysis. Baseline characteristics are presented as proportions and frequencies for categorical variables, and mean and corresponding standard deviations for continuous variables.

The incidence of AKI was expressed in person-time and was determined as the number of new cases who developed AKI during the study period. A sub-analysis of the patients with AKI was done. Bivariate and multivariate analysis was used to get risk ratios with their 95% confidence interval (CI) and P-values using modified Poisson regression analysis. Variables with a p-value of less than 0.2 at bivariate were further analysed at multivariate.

### Quality Control

The questionnaire was pre-tested on 10 patients who were not part of the study and any unclear statements were corrected

The two research assistants used were trained on how to use the questionnaire.

All samples analyzed for serum creatinine were done from the Mulago National Referral Hospital Laboratory. Any sample that was not analyzed immediately was temporarily stored at –20 < oC.

## Results

### Description of study participants

A total of 224 participants were recruited for this study, male: female ratio was 6.5:1 and the commonest reason for admission was due to road traffic accident (n=162, 72.3%). The mean age was 32±11 years, most participants (n=111, 49.6%) were peasants and 109 (48.7%) had attended primary school education as shown in Table 1.

**Table 1:** Baseline characteristics of the 224 study participants

Characteristic	Proportion (n %)
**Age**	
18–35	13 (5.8)
36–55	162 (72.3)
>55	49 (21.9)
**Sex**	
Female	30 (13.4)
Male	194 (86.6)
**Employment status**	
Student	16 (7.1)
Peasant	111 (49.6)
Formal employment	46 (20.5)
Boda-boda rider	51 (22.8)
**Mechanism of injury**	
Road traffic accident	162 (72.3)
Assault	47 (21.0)
Fall	15 (6.7)
**Type of injury**	
Penetrating injury	30 (13.4)
Blunt force	194 (86.6)

### Clinical characteristics

Most of the participants (n=105, 46.9%) presented to the hospital emergency unit after 24 hours of injury and more than half (n=136, 60.7%) were severely injured as shown in Table 2.

**Table 2:** Clinical characteristics of the 224 study participants

Characteristic	Proportion (n %)
**Time to presentation**	
<24 hours	51 (22.8)
12–24 hours	68 (30.3)
>24 hours	105 (46.9)
**Glasgow Comma Scale**	
Mild head injury	92 (41.1)
Moderate head injury	59 (26.3)
Severe head injury	73 (32.6)
**Injury Severity Score**	
<25	88 (39.3)
≥25	136 (60.7)
**Systolic BP at admission**	
Normal	161 (71.9)
High	63 (28.1)
**Random Blood sugar at admission in** **mg/dl**	
<79 (Hypoglycemia)	3 (1.3)
79–139 (Normal)	112 (50.0)
140–199 (Impaired)	72 (32.1)
≥200 (Hyperglycemia)	37 (16.5)

### Incidence of acute kidney injury

Acute kidney injury was found in 105/224 participants which translated to accumulative incidence of 46.9% (95%CI 40.2 – 53.6). The daily incidence was 67/1000 persons per day. There was a higher incidence of AKI among men, 43.3% (95%CI 36.7 – 50.1) compared to women 3.6% (95%CI 1.6–6.9).

### Demographic risk factors for acute kidney injury following major trauma

Participant age and sex were the risk factors that had a P<0.2 at bivariate and only sex remained significant at multivariate level as shown in Table 3.

**Table 3:** Demographic risk factors for acute kidney injury following major trauma

Characteristic	No AKI (n %)	AKI (n %)	Crude RR	P-value	Adjusted RR (95%CI)	P-value
**Age**						
18–35 (young adults)	8 (61.5)	5 (38.5)	1.00		1.00	
36–55 (middle aged)	89 (54.9)	73 (45.1)	1.17	0.662	1.20 (0.59–2.43)	0.619
>55 (older)	22 (44.9)	27 (55.1)	1.43	0.337	1.50 (0.73–3.10)	0.273
**Sex**						
Female	22 (73.3)	8 (26.7)	1.00		1.00	
Male	97 (50.0)	97 (50.0)	1.88	0.044	**2.37(1.17–4.89)**	0.017*
**Employment status**						
Student	10 (62.5)	6 (37.5)	1.00		1.00	
Peasant	51 (45.9)	60 (54.1)	1.44	0.275	1.29 (0.73–2.28)	0.376
Formal employment	28 (60.9)	18 (39.1)	1.04	0.909	1.16 (0.63–2.15)	0.637
Boda-boda rider	30 (58.8)	21 (41.2)	1.10	0.797	1.01 (0.54–1.90)	0.971
**Mechanism of injury**						
Fall	7 (46.7)	8 (53.3)	1.00		1.00	
Road traffic accident	86 (53.1)	76 (46.9)	0.88	0.617	0.89 (0.63–1.26)	0.509
Assault	26 (55.3)	21 (44.7)	0.84	0.544	1.01 (0.66–1.51)	0.988
**Type of injury**						
Penetrating injury	21 (70.0)	9 (30.0)	1.00		1.00	
Blunt force	98 (50.5)	96 (49.5)	1.65	0.083	1.30 (0.74–2.27)	0.361

### Clinical risk factors to AKI in major trauma patients

Time to presentation to the hospital, random blood sugar levels at admission in mg/dl, Glasgow Comma Scale and non-operative management were the factors with P<0.2 at bivariate analysis

### Outcomes

#### Survival probabilities of patients according to AKI as an outcome

There was no statistical difference in the survival probabilities of patients who developed AKI and those who did not over the 7 days follow-up period. However, survival of patients with AKI declined more from day 3 to day 7.

### Mortality rate

A total of 62 patients died during the study, giving an overall mortality of 27.7% (95%CI 21.9 – 30.0) and of these, 34 (54.8%) patients had AKI. The overall mortality rate during the study period was 39.5 patients per thousand per day.

## Discussion

In this study we set out to determine the incidence and risk factors of acute kidney injury among patients with major trauma in Mulago Hospital.

In our study, there was a high incidence of AKI of 67/1000 persons per day as measured using the serum creatinine criterion. This means every day approximately 7 out of 100 persons admitted with major trauma at Mulago hospital get AKI. The cumulative incidence was also high at 46.9 % (95%CI 40.2 – 53.6). This result might be due to rhabdomyolysis from trauma and the fact that most of the patients (105/224) presented after 24 hours. Rhabdomyolysis has been reported to cause acute renal failure in patients with trauma^13^–^15^. Similar findings have been reported in United Kingdom^16^,^17^, Zagazig University Hospital in Egypt^18^ and in France^19^.

In this study, presenting to hospital after 12 hours of injury was associated with an increased risk of developing AKI. Patients who presented between 12 to 24 hours of injury had 76% increase in the risk of developing AKI compared to those who presented earlier. Similarly, patients who presented after 24 hours from injury had a 67% increase in the risk of developing AKI compared to those who presented before 12 hours (table 4). This means AKI was more likely to develop in patients who presented late to hospital after a major trauma. This result may be due to kidney stress from volume depletion due to blood loss and rhabdomyolysis.

**Table 4:** Clinical risk factor to AKI in major trauma patients

Characteristic	No AKI (n %)	AKI (n %)	Crude RR	P-value	Adjusted RR	P-Value
**Time to presentation**						
<12 hours	34 (66.7)	17 (33.3)	1.00		1.00	
12–24 hours	34 (50.0)	34 (50.0)	1.50	0.081	**1.76 (1.12–2.77)**	0.014*
>24 hours	51 (48.6)	54 (51.4)	1.54	0.049	**1.67 (1.09–2.57)**	0.019*
**Glasgow Comma Scale**						
Mild head injury	55 (59.8)	37 (40.2)	1.00		1.00	
Moderate head injury	31 (52.5)	28 (47.5)	1.18	0.377	1.13 (0.79–1.63)	0.493
Severe head injury	33 (45.2)	40 (54.8)	1.36	0.063	1.16 (0.82–1.63)	0.402
**Injury Severity Score**						
<25	48 (54.5)	40 (45.5)	1.00		1.00	
≥25	71 (52.2)	65 (47.8)	1.05	0.734	1.01 (0.83–1.46)	0.514
**Systolic BP at admission**						
Normal	88 (54.7)	73 (45.3)	1.00		1.00	
High	31 (49.2)	32 (50.8)	1.12	0.454	0.90 (0.67–1.21)	0.485
**Random Blood sugar at** **admission in mg/dl**						
>79 (Hypoglycemia)	0	3 (100)	2.29	<0.001	**1.62 (1.24–2.12)**	**<0.001**
79–139 (Normal)	63 (56.3)	49 (43.7)	1.00		1.00	
140–199 (Impaired)	39 (54.2)	33 (45.8)	1.05	0.781	0.98 (0.70–1.38)	0.920
≥200 (Hyperglycemia)	17 (45.9)	20 (54.1)	1.24	0.256	1.23 (0.84–1.77)	0.276
**Operative management**						
Yes	59 (58.4)	42 (41.6)	1.00		1.00	
No	60 (48.8)	63 (51.2)	1.23	0.158	**1.39 (1.02–1.89)**	0.038*
**Received antibiotics**						
No	10 (50.0)	10 (50.0)	1.00		1.00	
Yes	109 (53.4)	95 (46.6)	0.93	0.764	0.92 (0.56–1.51)	0.739
**Received steroids**						
No	114 (53.0)	101(47.0)	1.00		1.00	
Yes	5 (55.6)	4 (44.4)	0.95	0.884	0.97 (0.46–2.03)	0.931
**Received NSAIDS**						
No	116 (53.5)	101(46.5)	1.00		1.00	
Yes	3 (42.9)	4 (57.1)	1.23	0.542	1.05 (0.50–2.21)	0.899

In this study, presenting with hypoglycemia (RBS <79mg/dl) on admission was associated with a 62% increase in the risk of developing AKI (table 4). This is because of early hypoglycemia that happens in multiple trauma patients with increased metabolic demands and this has been shown to be associated with developing and worsening of AKI^20^,^21^. Another explanation could be pre-existing diabetic disease or use of hypoglycemic drugs. This result is in agreement with what was reported in Baylor College of Medicine, Houston, Texas^22^.

**Figure 1:**
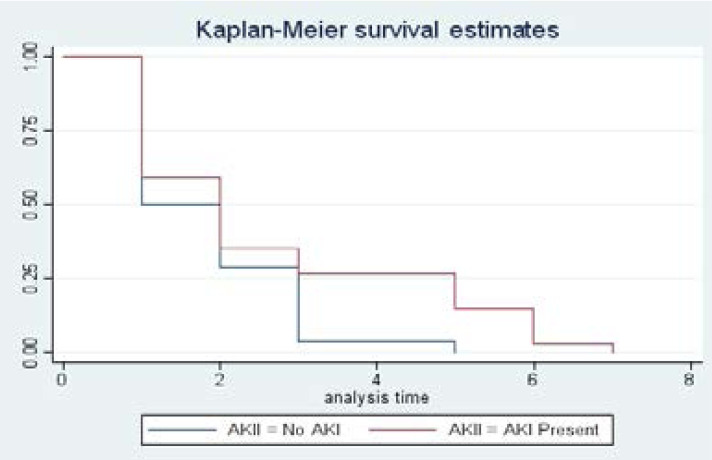
Survival curve among patients who developed AKI and those who did not

In this study, patients with major trauma who had non operative management had a 39% increase in the risk of developing AKI (table 4). This could be due to the differences in resuscitation among patients who underwent surgery and those who did not as this included restoration of intravascular volume which improved tissue perfusion. We found out that, of the 62 patients that died during the study, more than half, 34 (54.8%) had AKI. Survival of the latter declined more from day 3 to day 7 (figure 1). It is possible that these patients developed AKI related complications like uremic encephalopathy, uremic pericarditis, and electrolyte and acid-base disturbances. Similar studies have also reported higher mortalities in trauma patients with AKI patients^19^, ^20^

## Study limitations

The follow up duration of the AKI group should have been at least three months to help know those who developed chronic kidney disease.

## Recommendations

Close monitoring of kidney function in trauma patients must be routinely done.

## Conclusions

Acute kidney injury increases mortality in trauma.

## Data Availability

Data sets are readily available on request
